# Child/Adolescent’s ADHD and Parenting Stress: The Mediating Role of Family Impact and Conduct Problems

**DOI:** 10.3389/fpsyg.2017.02252

**Published:** 2017-12-22

**Authors:** Alicia Muñoz-Silva, Rocio Lago-Urbano, Manuel Sanchez-Garcia, José Carmona-Márquez

**Affiliations:** ^1^Department of Social, Developmental and Educational Psychology, University of Huelva, Huelva, Spain; ^2^Department of Psychology, University of Cádiz, Cádiz, Spain; ^3^Department of Clinical and Experimental Psychology, University of Huelva, Huelva, Spain

**Keywords:** parenting stress, child/adolescent’s ADHD, conduct problems, emotional problems, family impact, perceived social support, mediation models

## Abstract

**Objective:** The demands of parenting are usually associated with some stress, and elevated levels of stress may affect the parent–child relationships and parenting practices. This is especially the case of families where children have special needs conditions or disorders, like Attention Deficit Hyperactivity Disorder (ADHD).

**Method:** This study examined parenting stress among mothers of children and adolescents with ADHD. The sample comprised 126 mothers of girls (36; 29%) and boys (90; 71%) aged 6–17 years old.

**Results:** Mothers reported their own stress levels as well as the children and adolescents’ variables (severity of their ADHD symptoms, conduct, and emotional problems) and family–contextual variables (negative impact on family’s social life, impact on couple relationship, and perceived social support). Hierarchical multiple regression showed that (a) negative impact on social life and conduct problems were the strongest predictors of mother’s stress. Bootstrap mediation analyses revealed that (b) the association between child and adolescent’s ADHD and parenting stress was mediated by children’s conduct problems and by negative impact on family’s social life, and not by children’s emotional problems nor by mother’s perceived social support. The mediation analysis also suggested (c) a pathway from child/adolescent’s ADHD through children’s conduct problems and then through their negative impact on family’s social life to mother’s parenting stress.

**Conclusion:** These results suggest that both child/adolescent’s and family factors should be considered in the designing of interventions for reducing parenting stress in families of children and adolescents with ADHD.

## Introduction

Attention deficit hyperactivity disorder (ADHD) is a chronic and pervasive condition that begins in childhood and is characterized by inattention, impulsivity, and hyperactivity (American Psychiatric Association, 2013). ADHD usually leads to difficulties of adaptation in family environment, school, and in the relationships with peers. The ADHD prevalence is controversial. Thus, for example, whereas Polanczyk et al. (2007) found a pooled prevalence of 5% for subjects 18 years of age or younger, Thomas et al. (2015) found a prevalence of 7% in non-clinical children and adolescents’ samples. In Spain, the prevalence among children and adolescents has been estimated to be from 5% (Informe PANDAH, 2015) to 6.8% (Catalá-López et al., 2012).

The effects of ADHD are not confined to the individual alone, but go beyond and can affect his/her immediate social context, especially the school and family context. In the school setting, the student–teacher relationship was significant in preventing students’ risk of school failure or hyperactive–impulsive behaviors both in typically developed children (Longobardi et al., 2016a,b) and ADHD children (Rogers et al., 2015; Prino et al., 2016). In the family context, on the other hand, ADHD is commonly associated with elevated levels of parenting stress because the parents’ perceptions of the demands of their role as parents exceed their resources to cope with them.

Several works have been carried out on the bidirectional relationship (parent-to-child and child-to-parent processes) between parenting stress and child/adolescent’s ADHD symptoms: some studies point out that family problems (such as parental stress) can increase both the symptoms of ADHD and the psychological maladjustment of children (Deault, 2010; Haack et al., 2014). Probably, the increased parental stress leads to negative parenting styles (e.g., inconsistent discipline, and corporal punishment) which reinforce the unsuitable conducts of children (Patterson, 2002; Choe et al., 2013). However, a larger number of investigations have focused on the extent to which the behaviors of children with ADHD generate elevated levels of anxiety or parental stress (Donenberg and Baker, 1993; Podolski and Nigg, 2001; Theule et al., 2011, 2013), or even have shown that the children’s problems affect the parenting stress more than parenting stress affects the children’s problems (Mackler et al., 2015). This work fits within this last approach.

In this sense, several studies have shown that parents of ADHD children/adolescents experienced more parental stress than mothers and fathers of control children (Pimentel et al., 2011; Cussen et al., 2012; Wiener et al., 2016). Furthermore, the parents of children with ADHD reported greater parenting stress than parents of children with serious conditions as Epilepsy (Gagliano et al., 2014) or Autism Spectrum Disorder (Miranda et al., 2015). This relationship is not explained only by the ADHD symptoms themselves, but it is also due to the different problems comorbid with child ADHD such as oppositional defiant disorder (Sollie et al., 2016) or learning disabilities (Li et al., 2016), among other variables, some of which are analyzed in this paper.

According to the Abidin’s (1990, 1992) model, parenting stress is explained by both parent and child characteristics and situational variables. The influence of parents’ characteristics on parenting stress has been widely documented. Several investigators have shown to be associated with parenting stress the mothers and fathers’ trait anxiety (Delvecchio et al., 2015), the maternal depression (van der Oord et al., 2006), or the parents ADHD symptoms (Theule et al., 2011). Apart from child/adolescent ADHD symptoms and parents’ characteristics, the literature on parenting stress identified other relevant variables: presence of child/adolescent’s externalizing and/or internalizing problems, parents’ low marital quality, impact of the children ADHD symptoms in family life, and low levels of parental perceived social support (see, e.g., the exhaustive review of parenting stress and ADHD by Theule et al., 2013). The present study analyzed the influence of child/adolescent and family/contextual variables on parenting stress. The age of the children may affect the course of the disorder and how it is perceived by adults (Olson, 2002; Monuteaux et al., 2010; Martel et al., 2016). However, due to the findings about the relationship between children’s age and parenting stress are generally inconsistent (Theule et al., 2013) or could change between boys and girls (Sollie et al., 2016), we consider it may be useful to use a wide range of age – children and adolescents together.

Among the determinants of parental stress in the Abidin’s (1990, 1992) model (parents, children, and family/contextual factors), we studied the last two factors. We used an ecological and systemic approach (Bronfenbrenner and Morris, 2006; Parke and Buriel, 2006) in which the parental stress is related to children’s characteristics (mainly the severity of ADHD symptoms, and comorbid conduct and emotional problems), and to family and contextual factors (the impact of the ADHD symptoms on family and on social support).

Child/adolescent problems. In relation to the severity of symptoms of ADHD, it is well established that the higher ADHD symptom severity, the more parenting stress (see, e.g., Anastopoulos et al., 1992; Healey et al., 2011; Theule et al., 2013). Moreover, common comorbid problems in children and adolescents with ADHD may negatively impact parent–child relationships and result in higher levels of parenting stress (e.g., Anthony et al., 2005). This is especially the case for the presence of externalizing behavior (Anjum and Malik, 2010; Bauermeister et al., 2010; Pimentel et al., 2011; Theule et al., 2011, 2013; Wiener et al., 2016). Furthermore, some studies also have underlined the influence of internalizing symptoms (Anjum and Malik, 2010; Graziano et al., 2011; Pimentel et al., 2011; Theule et al., 2013). In addition, some studies found that emotional reactivity/lability (internalizing) and aggression (externalizing) mediated the relation between child ADHD and parenting stress (e.g., Graziano et al., 2011).

Family and contextual variables. Both children ADHD (and comorbidities) and parental stress have been associated to family functioning variables and perceived social support. First, it has been well-documented the strong relationships between family dysfunction and child ADHD (Donenberg and Baker, 1993; Lange et al., 2005; Bauermeister et al., 2010; Sollie et al., 2016). High levels of parents’ disagreement experienced in regard to the child problems, low participation in social events, and high affectation of the family’s social life was related to high levels of child ADHD severity or/and conduct problems (Shelton et al., 1998; Fleck et al., 2015). These family variables, in turn, can affect the parent–child interactions and increase the parental stress. For example, higher levels of parental stress are founded for women and men who report lower satisfaction with their couple (Deater-Deckard, 2004; Norlin and Broberg, 2013) or greater restriction in their social activities (Cramm and Nieboer, 2011).

Second, several works have shown the powerful association of perceived social support with both parental styles and child/adolescent ADHD symptoms (or conduct problems): Families of children with ADHD perceive more social isolation and report lower perceive social support than control families (Lange et al., 2005; Gau, 2007). This is remarkably important considering that child/adolescent’s conduct problems augmented when perceived social support diminished (Mash and Johnston, 1983; Akcinar and Baydar, 2016). The role of social support as a stress-related factor has also been investigated. Some studies have shown that social support is inversely related to and is an important predictor of the levels of stress experienced by mothers (Weiss, 2002). The perception of social support has been identified as a critical parental resource for lowering parenting stress (Crnic and Greenberg, 1990; Solem et al., 2011) and for moderating and reducing the negative impact of the stress (Turner and Turner, 1999).

Finally, previous research has found relationships between both child/adolescent problems and family/contextual variables. For example, Armstrong et al. (2015) have shown longitudinal associations between baseline internalizing and externalizing problems and poor family quality of life at follow-up.

The main objective of the present study was to identify the predictors of parenting stress in a sample of families with children and adolescents with ADHD. We examined how the family and contextual factors contribute to the prediction of parenting stress after controlling for the effects of the children’s characteristics. This model would be consistent with the above-mentioned research indicating that both child/adolescent and family–contextual variables have predictive capacity. If the model is correct, then both child and family–contextual variables should be targeted for simultaneous improvement in the treatment of parental stress.

Despite Abidin’s (1992) mediation model posits that associations between parenting stress and child problems may be indirect, much of the attention of researchers has focused on the direct effects of child and family–contextual factors on parenting stress. But, from a practical and theoretical viewpoint, it is important to identify the processes that intervene between a risk factor such as child/adolescent ADHD and an outcome such as parental stress. It is relevant to increase our knowledge about these processes in order to base intervention programs for reducing parenting stress. In the present study, we consider the increase of emotional–conduct problems, the reduction of mother’s perceived social support, and the negative effects of ADHD in family’s social life and marital relationship as possible mechanisms through which child/adolescent ADHD severity is related to parental stress. The study of these potential mechanisms may inform approaches to change the pathway from ADHD to parenting stress. Specifically, we tested the following mediation hypotheses:

(A) The conduct and emotional problems of children may mediate the relation between child ADHD severity and parenting stress. According to the scientific literature about child/adolescent ADHD severity and parenting stress (see, e.g., Graziano et al., 2011; Theule et al., 2013; Wiener et al., 2016), the severity of ADHD is expected to have a direct effect on parenting stress and an indirect effect through its relationships with conduct and emotional problems.
(B) Following the approach of several researchers (e.g., Lange et al., 2005; Cramm and Nieboer, 2011; Solem et al., 2011; Fleck et al., 2015) we hypothesized that the family and contextual factors may mediate the relation between child ADHD severity and parenting stress. It thus may be the case that increased ADHD severity predicts higher negative impact on family and lower perceived social support, which may, in turn, be linked to higher levels of parenting stress.
(C) Additionally, according to the work of Armstrong et al. (2015) we explored a serial multiple mediator model between child/adolescent’s ADHD and parenting stress that included a direct path from children’s conduct and emotional problems to family and contextual factors. We hypothesized that one of the mechanisms by which ADHD is linked to mothers’ stress is through the effects of the emotional and behavioral difficulties experienced by children with ADHD on context and family functioning.

## Materials and Methods

### Participants

The sample included 126 mothers of children/adolescents diagnosed with ADHD aged 6–17 years. **Table 1** shows the main characteristics of the participants. As shown in the table, most children have the diagnosis of combined or inattentive subtype. Eighteen percent of the families did not know the subtype. Among the associated problems, it should be mentioned that almost one-third of the children and adolescents had learning disorders.

**Table 1:** Socio-demographic and clinical variables of children/adolescents.

Variable	*M*	*SD*	*N*	%
(1) Age	10.90	3.06		
(2) Gender: boys			90	71
(3) Subtype or presentation of ADHD				
Combined			46	37
Inattentive			43	34
Hyperactive/impulsive			11	9
Not answer			22	18
**Comorbidity: associated problems**				
(4) Learning disorders			39	31
(5) Oppositional defiant disorder			24	19
(6) Speech or expression disorders			19	15
(7) Anxiety			17	14

*ADHD, attention deficit hyperactivity disorder.*

The mean age of mothers was 42.84 years (*SD* = 7.36; range 24–73), and 115 (91%) were married or living with a partner. Regarding education level, 92 (73%) mothers had basic or secondary studies and 34 (27%) graduate or post-graduate studies.

### Procedure

To access families, the researchers visited the associations of families of children and adolescents with ADHD and the centers of educational assistance in Huelva (Spain). The professionals of these centers asked the voluntary collaboration of the parents in the study. All the participants filled their written informed consent. The bioethics committee on human research of the University of Huelva approved the study protocol.

Mothers provided all the information, completing self-report questionnaires about sociodemographic variables, children/adolescents characteristics, diseases and comorbid problems, and family and contextual variables. Child/adolescent ADHD was diagnosed by a professional pediatrician or psychologist in the above referred educational centers or family associations, in accordance to DSM-IV and Health Authority criteria (Ministerio de Sanidad, Política Social e Igualdad, 2010).

Out of the 184 mothers contacted to participate 140 completed the questionnaires (76% rate of response). Fourteen mothers were excluded from the analyses because their sons/daughters were 19 years or older. The children/adolescents whose mothers participated in the research had similar characteristics to those participating in other Spanish ADHD studies (Catalá-López et al., 2012; Informe PANDAH, 2015), especially in the boys/girl ratio and in the most common comorbid problems.

### Measures

#### Overall Parental Stress (OPS)

We used a scaled score of parenting stress obtained by equating the following two measures.

(A) Parenting stress index (PSI/SF; until 11 years-old) (Abidin, 1995; Spanish version by Díaz-Herrero et al., 2010, 2011). This self-report measure was administered to mothers of children aged less than 12 years. The brief version – 36 items, with 5-point Likert scale, ranging from “Strongly Disagree” to “Strongly Agree” – was applied. The PSI/SF produces scores on three subscales (Parental Distress, e.g., “Since having this child, I have been unable to do new and different things” –, Parent–Child Dysfunctional Interaction – “My child almost never does things that make me feel good” –, and Difficult Child – “I feel that my child is very moody and easily upset –”) that are added to yield an overall PSI. Regarding the reliability analysis, overall PSI demonstrates a good internal consistency in our sample (Cronbach’s α = 0.91). The Spanish version of the PSI/SF has been demonstrated to have adequate psychometric properties (e.g., α = 0.90, Díaz-Herrero et al., 2011; α = 0.92 in a mothers’ sample, Oliva et al., 2014).
(B) Stress index for parents of adolescents (SIPA, 12+ years old) (Sheras et al., 1998; Spanish Translation by Psychological Assessment Resources). It is a 90-item questionnaire filled by parents of adolescents that reflects the experienced parenting stress through three domains: adolescent (e.g., “My child has sudden changes of feelings or moods”), parent (e.g., “I feel alone and without friends”), and the adolescent–parent relationship (e.g., “My child comes to me for help more than to other people”). It also provides a measure of total parenting stress. Items are rated on a 5-point rating scale from 1 “Strongly Disagree” to 5 “Strongly Agree” (Cronbach’s alpha = 0.96, by the overall SIPA in this study). The scores of the Spanish version have good internal consistency (e.g., Sanchez-Sandoval and Palacios, 2012, α = 0.96).

In the creation of OPS scores we used an equipercentile equating method (Kolen and Brennan, 2014). We choose this method because of: (A) the two measures correspond to the same construct and theoretical model, and comprise almost the same dimensions; (B) their reliabilities are similar; (C) they follow an approximately normal distribution (Kolmogorov–Smirnov’s *Z*: PSI = 0.701; SIPA = 0.817); and (D) the authors provide information about how to convert raw scores to percentile rank-scores for each test (Abidin, 1995; Sheras et al., 1998). Those scores with the same percentile rank on the PSI and SIPA are considered equivalent. The equated scores were standardized and converted to *T*-scores. These standardized *T*-scores (OPS) allow the comparison of parents’ stress levels independently of the stress measure used.

#### Child/Adolescent ADHD Severity (C.ADHD)

The parent form of the Conners’ ADHD Index (Conners, 1997; Spanish version by Martínez et al., 2013) includes both inattention items (e.g., “Easily distracted,” “Inattentive”) and hyperactivity–impulsivity items (“Restless,” “Interrupts others”). It consists of 10 items; each rated on a four-point scale from 0 “Not at all” to 3 “A lot” (Cronbach’s α = 0.83 in this sample). The more symptoms and severity, the higher score on the scale. Reliability and validity evidence have been established for the Spanish version (e.g., Salas et al., 2015; α = 0.92).

#### Child/Adolescent Conduct Problems (C.CP) and Child/Adolescent Emotional Problems (C.EP)

The Strengths and Difficulties Questionnaire (SDQ; Goodman, 2001, Spanish version available at http://www.sdqinfo.com/py/sdqinfo/b3.py?language=Spanish) is a mental health screening questionnaire for children and adolescents with 25 items across five dimensions. For this study we used only two dimensions: the emotional symptoms (five items scoring from 0 to 2; e.g., “Often he is unhappy, discouraged or tearful”; Cronbach’s α = 0.68) and the conduct problems (five items scoring from 0 to 2; e.g., “He/she gets very angry and often loses his/her temper”; Cronbach’s α = 0.70) subscales since they are the most representative for internalizing symptoms and externalizing behaviors, respectively. Some studies have shown an adequate reliability of the Spanish version (α = 0.71 for emotional symptoms and α = 0.62 for conduct problems, Rodríguez-Hernández et al., 2012; and α = 0.70 and α = 0.74, respectively, Gómez-Beneyto et al., 2013).

#### Impact on Social Life Scale (Donenberg and Baker, 1993 Spanish Adaptation by Presentación-Herrero et al., 2006)

It is a 10-items 0–10 score scale. The lower participation in social events and more negatively influenced the family’s social life, the higher the score on the scale [e.g., “My family avoids social outings more (e.g., restaurants, public events) because of his/her behavior,” “I have guests over to our house less often than I would like to because of my child’s behavior”). The Cronbach’s a was 0.79 in the current study.

#### Impact on Marriage Scale (Donenberg and Baker, 1993)

Spanish adaptation by Presentación-Herrero et al., 2006). It is a 10-items 0–10 score scale. The higher levels of parents’ disagreement experienced in regard to the child problems, the higher the score on the scale (e.g., “My child causes more disagreements between my spouse and me”). The Cronbach’s a was 0.78 in the current study. The Spanish version of theses scales has adequate psychometric properties (Presentación-Herrero et al., 2006; Presentación et al., 2009).

#### Perceived Social Support (PSS)

The Multidimensional Perceived Social Support Scale (MPSSS; Zimet et al., 1988; Spanish adaptation by Landeta and Calvete, 2002). This scale has 12 seven-point Likert-type items, with responses from 1 “Very Strongly Disagree” to 7 “Very Strongly Agree.” The MPSSS evaluates the perceived support from friends, the family support, and the support from especially relevant persons. Total score varies from 12 to 84, and higher scores indicate higher levels of social support (Cronbach’s α = 0.92 in our sample). As examples of items: “I get the emotional help and support I need from my family” or “I can count on my friends when things go wrong.” Diverse studies have demonstrated its adequate internal consistency (α = 0.89, Buesa and Calvete, 2013 and α = 0.89, Landeta and Calvete, 2002).

### Data Analysis

We analyzed data using SPSS 20. After checking for outliers (using SPSS box-plot and Mahalanobis distance for univariate and multivariate analysis, respectively) and missing data, we analyzed the means, standard deviations, skewness and zero-order correlations of research variables. We also examined the associations of parenting stress with demographics and clinical variables to select control variables for further analyses. Also, we examined the regression assumptions of linearity, normality, and homoscedasticity by graphical inspection of residuals plots (scatter plots of standardized residuals vs. the predicted values and *Q*–*Q* plots). We found no serious violation of the regression assumptions.

We conducted a hierarchical regression analysis to explore the predictive power of child ADHD on mothers’ parental stress. We followed a proximal–distal sequence of additive effects, starting with ADHD severity, continuing with child problems associated with ADHD (conduct and emotional problems) and ending with family and contextual effects related to ADHD (mothers’ perceived social support and ADHD impact on marriage and social life). At each step in the regression analysis, the change in *R*-square was used as an indicator of the predictive power of each group of predictors when previous predictors were taken into account. A *post hoc* analysis was conducted reversing the two last steps in the regression model.

The hypotheses of mediation were evaluated using two parallel multiple mediator models (hypotheses A, B. Model 4 in Hayes, 2013) and a serial multiple mediator model (hypothesis C. Model 6 in Hayes, 2013) using parenting stress as the dependent variable, children ADHD as the independent variable, and family–contextual variables and children problems as the mediators. All variables were standardized prior to analyses. The direct and indirect effects were estimated using SPSS PROCESS macro (Hayes, 2013). We used a bootstrapping process (10,000 resamples) to estimate the 95% bias-corrected confidence intervals (95% BC CIs) of the indirect effects. An indirect effect was statistically significant if the interval did not include zero.

There was less than 1.5% missing data. A Little’s Missing Completely At Random (MCAR) tests was conducted (χ^2^ = 31.71, *df* = 34, *p* = 0.581), indicating no systematic missingness. Imputation for the bootstrap procedure was conducted using SPSS Multiple Imputation Procedures (i.e., expectation maximization algorithm). We applied SPSS PROCESS macro on the five groups of multiple imputed data. The average of the bootstrap indirect effects (and CI) across the five group was computed. On the other hand, our sample size was sufficient to detect mediated effects [power = 0.80 and small-to-medium paths: α = 0.26 and β = 0.39, according to Fritz and MacKinnon’s (2007) criteria].

## Results

### Preliminary Analyses

First, we analyzed the relationships between parenting stress and the socio-demographic and clinical variables presented in **Table 1**. Results showed no significant relationships, except for children’s age (*r* = -0.22, *p* = 0.02) and mother’s age (*r* = -0.20, *p* = 0.03). Thus, children/adolescents’ age and mothers’ age were added to the regression models and were used as covariates in the mediation models.

### Hierarchical Regression Models

**Table 2** shows the intercorrelations among study variables. The pattern of correlations was consistent with expectations. Parental stress was positively correlated with children ADHD severity, conduct, and emotional problems and with mothers’ perceptions of ADHD impact on marriage and social life, and negatively correlated with mothers’ perceptions of social support. A similar pattern of correlations was observed for child ADHD severity. On the other hand, whereas ADHD impact on social life was strongly correlated with child/adolescent emotional and conduct problems, ADHD impact on marriage and mothers’ social support was unrelated to child/adolescent emotional problems and only slightly related to conduct problems. Also noteworthy are the negative correlations between mothers’ social support and ADHD impact on marriage and social life.

**Table 2:** Means, standard deviations, observed range, and correlations among study variables.

	OPS	C.ADHD	C.EP	C.CP	SOC	MAR	PSS
OPS	1.00						
C.ADHD	0.46^∗∗∗^	1.00					
C.EP	0.33^∗∗∗^	0.41^∗∗∗^	1.00				
C.CP	0.46^∗∗∗^	0.52^∗∗∗^	0.37^∗∗∗^	1.00			
SOC	0.49^∗∗∗^	0.46^∗∗∗^	0.32^∗∗∗^	0.46^∗∗∗^	1.00		
MAR	0.30^∗∗^	0.25^∗∗^	0.16	0.23^∗^	0.38^∗∗∗^	1.00	
PSS	–0.35^∗∗∗^	–0.29^∗∗^	–0.12	–0.23^∗^	–0.39^∗∗∗^	–0.37^∗∗∗^	1.00
Mean	63.15	16.36	4.07	3.97	2.40	2.32	62.99
*SD*	9.60	5.53	2.41	2.45	2.85	2.11	14.60
Observed range	39.64–75.89	2–28	0–10	0–10	0–10	0–7	20–92
Skewness	–0.678	–0.160	0.387	0.411	1.181	0.775	–0.737

*OPS, overall parenting stress; C.ADHD, child/adolescent ADHD severity; C.EP, child/adolescent emotional problems; C.CP, child/adolescent conduct problems; SOC, impact on social life; MAR, impact on marriage; PSS, perceived social support. ^∗^*p* < 0.05; ^∗∗^*p* < 0.01; ^∗∗∗^*p* < 0.001.*

**Table 3** shows the results of the hierarchical multiple regression predicting mothers’ stress as a function of child and family–contextual factors, controlling for child/adolescent’s and mother’s age, entered in the first step. Child ADHD severity was entered in the second step, followed by children conduct and emotional problems in the third step, and family–contextual variables in the final step. Children’s and mother’s age explained the 7% of the parenting stress variance, but only child/adolescent’s age had a slight weight. Child ADHD severity accounted for a significant amount of the parenting stress variance over and above that explained in the first step (Δ*R*^2^ = 0.17, *p* < 0.001): mothers of children with higher levels of ADHD had higher levels of stress.

**Table 3:** Hierarchical regression model for predicting parenting stress.

Models	β	*R*^2^	Δ*R*^2^
Step 1		0.07	0.07^∗^
Child/adolescent’s age	–0.17^+^		
Mother’s age	–0.14		
Step 2		0.24	0.17^∗∗∗^
Child/adolescent’s age	–0.14		
Mother’s age	–0.07		
C.ADHD	0.43^∗∗∗^		
Step 3		0.35	0.11^∗∗∗^
Child/adolescent’s age	–0.21^∗^		
Mother’s age	–0.08		
C.ADHD	0.19^∗^		
C.EP	0.12		
C.CP	0.35^∗∗∗^		
Step 4		0.44	0.09^∗∗^
Child/adolescent’s age	–0.26^∗∗^		
Mother’s age	–0.04		
C.ADHD	0.08		
C.EP	0.11		
C.CP	0.26^∗∗^		
SOC	0.20^∗^		
MAR	0.12		
PSS	–0.13		
		Total *R*^2^ = 0.44^∗∗∗^	

*C.ADHD, child/adolescent ADHD severity; C.EP, child/adolescent emotional problems; C.CP, child/adolescent conduct problems; SOC, impact on social life; MAR, impact on marriage; PSS, perceived social support. ^+^*p* = 0.066; ^∗^*p* < 0.05; ^∗∗^*p* < 0.01; ^∗∗∗^*p* < 0.001.*

The third step demonstrated that other child factors, especially child conduct problems, explained a significant amount of parental stress variance over and above that explained by the ADHD severity (Δ*R*^2^ = 0.11, *p* < 0.001). The inclusion of contextual–family variables in the fourth step accounted for an additional significant 9% of the variance in mothers’ stress (Δ*R*^2^ = 0.09, *p* < 0.01). The only significant predictors of mothers’ stress in the final model (controlling for children’s and mother’s age) were the child conduct problems and the ADHD impact on family’s social life. The more negative the effects on social life, and the more child/adolescent conduct problems, the higher the levels of parental stress. When we reversed the order of entry in the regression model, entering the family and contextual variables in the third step, the child factors significantly predict parenting stress over and above family and contextual variables (Δ*R*^2^ = 0.06, *p* < 0.01, in the fourth step).

### Mediation Models

**FIGURE 1:**
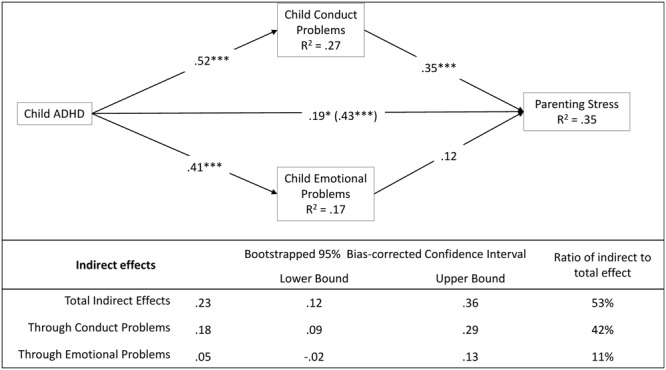
The mediational role of child/adolescent conduct and emotional problems. Coefficients are standardized estimates (total effect in bracket). Using children/adolescents’ age and mothers’ age as covariates. ^∗^*p* < 0.05; ^∗∗^*p* < 0.01; ^∗∗∗^*p* < 0.001.

First, we tested whether the effects of child ADHD severity on parental stress were mediated by other child factors – conduct and emotional problems (**Figure 1**). Results showed that child ADHD severity was significantly related to child/adolescent conduct and emotional problems. Moreover, the total effect of ADHD on parenting stress (*c* = 0.43, *p* < 0.001) can be decomposed into direct and indirect effects. The indirect effect of child ADHD severity through child conduct problems was significant and suggest that ADHD predict the parenting stress through its effect on conduct problems (*a*_1_*b*_1_ = 0.18, *p* < 0.05): the existence of a higher number of conduct problems partially explain why mothers of children/adolescents with more severe ADHD were more likely to report higher rates of stress. This mediation was only partial and the child ADHD severity also had a significant direct impact on mothers’ stress (*c’* = 0.19, *p* < 0.05).

**FIGURE 2:**
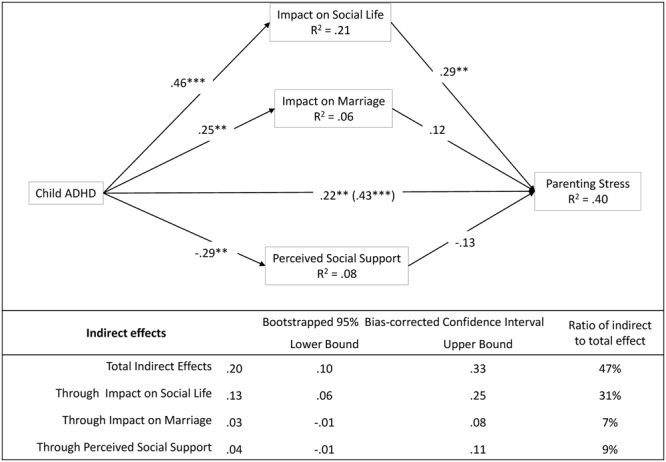
The mediational role of family and contextual factors. Coefficients are standardized estimates (total effect in bracket). Using children/adolescents’ age and mothers’ age as covariates. ^∗^*p* < 0.05; ^∗∗^*p* < 0.01; ^∗∗∗^*p* < 0.001.

The second parallel mediation model tested the indirect effect of ADHD severity on mothers’ stress through family–contextual factors (**Figure 2**). The child/adolescent ADHD severity had a significant impact on family’s social life, marriage, and perceived social support. However, impact on social life was the only one of these contextual factors with a mediating role in the relationship between ADHD severity and parental stress (*a*_1_*b*_1_ = 0.13, *p* < 0.05). In particular, the 31% of the total effect of child/adolescent ADHD severity on parental stress was mediated by the level of restriction of social life of the family. There was still a significant direct effect of ADHD severity (*c*′ = 0.22, *p* < 0.01) that accounted for a 53% of the variance of mothers’ stress in this model.

**FIGURE 3:**
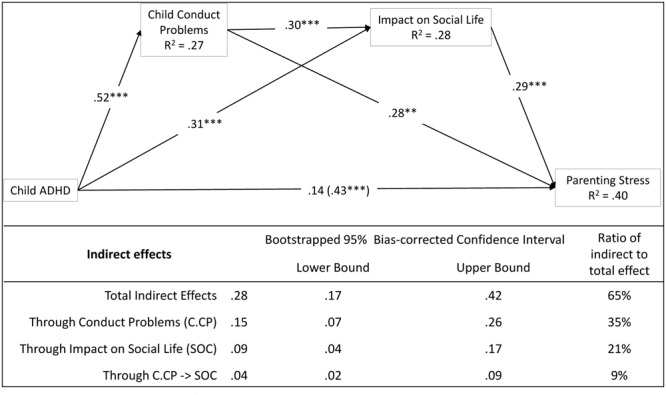
Child/adolescent problems predict parenting stress. The mediational role of family and contextual factors. Coefficients are standardized estimates (total effect in bracket). Using children/adolescents’ age and mothers’ age as covariates. ^∗^*p* < 0.05; ^∗∗^*p* < 0.01; ^∗∗∗^*p* < 0.001.

Finally, we tested whether the impact of ADHD severity on mothers’ stress was mediated by a serial process that goes from child/adolescent factors to family–contextual factors. Given the results of the former parallel mediation models, we proposed a serial mediation model with child conduct problems and ADHD impact on social life as mediators (**Figure 3**).

We found that all the three specific indirect effects in the model were significant, that is the effect of ADHD severity on stress (a) through child conduct problems (*a*_1_*b*_1_ = 0.15, *p* < 0.05), (b) through social life (*a*_2_*b*_2_ = 0.09, *p* < 0.05), and (c) through child conduct problems and ADHD impact on social life operating in serial (*a*_1_*d*_21_*b*_2_ = 0.04, *p* < 0.05). Together, these three indirect effects accounted for a 65% of the total effect. Moreover, the direct effect of ADHD severity on stress was nonsignificant in this model (*c*′ = 0.14, *p* = 0.13).

## Discussion

As it is clearly stated by Theule et al. (2013), the behavior of children and adolescents with ADHD implies great difficulties to parents for their education, generating high levels of family stress which affect parental physical and mental health and parenting practices. In order to reduce the stress of parents it is necessary to know the factors affecting parental stress. We found that (a) higher parenting stress was associated with higher levels of children/adolescents ADHD symptoms and conduct/emotional problems, higher interparental conflict/disagreement, higher disturbance of social life of family members, and lower levels of perceived social support; and (b) contextual factors predict parenting stress over and above the children/adolescents problems and vice versa. Beyond this, our study explores the interrelationships between child’s problems, contextual factors, and parenting stress, controlling for child/adolescent’s and mother’s age. Through three mediation models we stated that (c) conduct problems partially mediate the relationship between ADHD and parenting stress; (d) family’s social life partially mediate the relationship between ADHD and parenting stress; and (e) the relationship between conduct problems and parenting stress was mediated by the negative impact of child/adolescents’ problems on the family’s social relationships. We believe that, despite the abundant literature found on this topic, this work makes an important contribution. This paper provides additional information about the mediating role of family’s social life in the relationship between ADHD severity and parenting stress. Furthermore, is a novel contribution the serial multiple mediator model from child/adolescent ADHD symptoms to parenting stress with conduct problems as first mediator variable and impact in social family life as second mediator.

### Child/Adolescent’s Factors and Contextual Variables Predict Parenting Stress

The findings of our study show that parental stress is correlated to child/adolescent ADHD severity, emotional problems, conduct problems, impact on marriage and social life, and perceived social support. In our opinion, these findings enable a more detailed understanding of the complexities affecting parenting stress in families of children with ADHD. Our results support the view that parenting stress depends on both child and family/contextual factors (Theule et al., 2011, 2013). Thus, we can confirm the importance of child ADHD severity and child comorbid problems in the prediction of parental stress (Wiener et al., 2016). Moreover, regarding the influence of family and contextual factors on parental stress, we found data that support the role of family’s social life (Donenberg and Baker, 1993; Cussen et al., 2012; Moen et al., 2015).

The results of the hierarchical regression analyses revealed the significant predictive role of the family and contextual factors, even when we considered the child factors. Specifically, we found an increment of 9% of explained variance when we introduced the family and contextual factors in the model that predict the parental stress from the child/adolescent factors. Similarly, Anderson (2008) in a multiple regression model found that both child/adolescent behavior and family context (family cohesion and family involvement) significantly predict parenting stress. Finally, hierarchical regression models (controlling for children’s and mothers’ age) indicated that the main predictors of parental stress were child conduct problems and negative impact of ADHD in family’s social life. Therefore, parenting stress can be predicted more from the restriction or alteration of family’s social activities and from their child comorbid symptoms than from their perceived social support, their child/adolescent’s ADHD severity, or from the ADHD impact on their marriage relationships (McLaughlin and Harrison, 2006; Cramm and Nieboer, 2011).

### Conduct Problems and Impact on Family’s Social Life Mediate between ADHD Severity and Parenting Stress

Moreover, mediation analyses indicated that child ADHD severity had both direct and indirect effects on parenting stress. We found that child/adolescent ADHD severity can increase the child conduct and emotional problems. In turn, conduct problems can affect the parental stress. This mediating role of conduct problems is congruent with other studies (Graziano et al., 2011) in which children comorbid behavioral problems were perceived by parents as more stressful than ADHD symptoms. Probably, the parents’ concerns about their role in the development of behavior problems – but not about ADHD origin – may increase their levels of stress (Donenberg and Baker, 1993).

Finally, family functioning seems to play an important role in the prediction of parental stress. We discovered that the indirect effect of ADHD severity through its impact on family was almost equal than its direct effect. Therefore, it appears that the negative impact on family’s social life functioning is one of the main mechanisms of influence of child ADHD on mothers’ parental stress, more than the marital disputes or disagreements. Moreover, the multiple mediation model showed that the close relationship between conduct problems and parental stress is mediated by the negative impact these problems have on the social life of the family members. The association between child/adolescent conduct problems and mothers’ stress levels will increase as these problems affect the social life of the family. This finding has important implications for practice: parent-training programs should take in account the improvement of parents’ social life (e.g., allowing parents to regularly socialize with friends) to reduce parenting stress (Cramm and Nieboer, 2011).

On the other hand, we found that perceived social support did not play a relevant role in predicting the stress of parents. A possible explanation for this result may be that the relationship between perceived social support and parental stress was (also) mediated by family’s social life. This indirect effect could be explained if we consider that an important source of social support is the instrumental support – e.g., taking care of the child by relatives, friends, or acquaintances – that allows the parents to maintain social life, reducing indirectly the parental stress. Unfortunately, we could not examine the effect of distinct types of social support because we only used the total score of the social support scale. In this sense, Cohen et al. (1985) have shown that the perception of support only matters when the type of support matches the perceived need. In future researches will be interesting to use a more specific scale like the Family Support Scale (Dunst et al., 1984), a widely used parent self-report scale in the parenting stress studies (Theule et al., 2011; Woodman, 2014).

### Limitations

Several limitations of the present study should be mentioned: our study was cross-sectional, our sample was only mothers, and the sample size was limited. First, although the findings of this study suggest the mediating role of child/adolescent conduct problems or family’s social life, the cross-sectional design of this study limits any causal interpretation and precludes conclusions about the directionality of relationships. Despite some works have suggested that the effect of child problems on parenting stress is stronger than the opposite effect (Mackler et al., 2015), and despite that the structure and direction of our mediation models was based on past theoretical and empirical work, reverse effects to those demonstrated are possible. Future research should use longitudinal design (or experimental) to establish causal relations.

Second, the hierarchical regression and mediation models were only tested for mothers. This is a major limitation because the point of view of fathers was absent and the strength of the relations may have been inflated by this informant effect. Several studies analyzed the disagreement between informants and supported the value of multi-informant assessment (Biederman et al., 2004; Kerr et al., 2007; De los Reyes, 2011). Furthermore, some studies have shown that mothers seem to be more sensitive to their children’s behavior than fathers (e.g., Calzada et al., 2004), so their lives would be more altered. Recently, Delvecchio et al. (2015) and Wiener et al. (2016) also found different correlates of parenting stress for mothers and fathers. In a related research, Quittner et al. (1998) found that conflict over child rearing predicted father’s parental stress but not mother’s stress. Conflict over child rearing is a central concept in the measure of impact on marriage, which had no effect on mother’s stress in our study. Thus, despite that the primary attachment figure is usually the mother, futures studies should include multiple sources of information to verify the invariance of models across informants.

Third, given the limited sample size, we did not analyze the invariance of our mediation model by age or gender groups. Although there are some previous studies that pointed to the suitability of separate analyzes for children and adolescents (see Olson, 2002; Martel et al., 2016), the aggregation of results from a literature review (Theule et al., 2013) indicated no clear relationship between parental stress and children’s age. These conflicting results led us not to limit the range of ages in our study. Instead, we decided to control the effect of the age statistically. Furthermore, because some research have shown that children’s gender may have an impact on parental stress (e.g., Sollie et al., 2016) and on family functioning(Bauermeister et al., 2010), the effect of children’s gender should also taken into account in regression and mediation models. However, we acknowledged that sample size limitations make this analysis strategy suboptimal. Therefore, the results of our study should be further confirmed using larger samples that allow a more powerful study of gender- and age-specific effects.

### Implications

Despite these limitations, our study has several strengths and practical implications, and contributes to the extant literature. While most research has focused on the analysis of correlations or regression models with some selected variables, our work provides an overview of the relationships between family and contextual variables, child/adolescent problems, and parental stress. Based on our findings, we recommend that intervention in families of children with ADHD should provide parents with strategies for managing child behavior and relaxation skills to reduce their psychological and emotional stress and improve family well-being (Anastopoulos et al., 1993). On the other hand, our results support the development of family-oriented interventions programs to reduce parental stress, for example, promoting resilient behavior among parents facing social limitations to reach an active social life (such as maintaining valued social lives) (Emerson et al., 2010; Cramm and Nieboer, 2011).

## Author Contributions

All authors contributed to the interpretation of data, helped to draft and have revised the manuscript to get the final text.

## Conflict of Interest Statement

The authors declare that the research was conducted in the absence of any commercial or financial relationships that could be construed as a potential conflict of interest.
